# Outcome for triple negative breast cancer in a retrospective cohort with an emphasis on response to platinum-based neoadjuvant therapy

**DOI:** 10.1007/s10549-018-5066-6

**Published:** 2018-11-28

**Authors:** Elaine M. Walsh, Aliaa Shalaby, Mark O’Loughlin, Nessa Keane, Mark J Webber, Michael J. Kerin, Maccon M. Keane, Sharon A. Glynn, Grace M. Callagy

**Affiliations:** 1 Discipline of Pathology, Lambe Institute for Translational Research, NUI Galway, Costello Road, Galway, Ireland; 2 Department of Medical Oncology, University Hospital Galway, Galway, Ireland; 3 Discipline of Surgery, Lambe Institute for Translational Research, NUI Galway, Costello Road, Galway, Ireland

**Keywords:** Triple negative, Breast cancer, Survival, Pathological complete response, Neoadjuvant, Carboplatin

## Abstract

**Purpose:**

The rate of pathological complete response (pCR) for patients with triple negative breast cancer (TNBC) is increased when carboplatin is added to neo-adjuvant chemotherapy (NACT). However, while phase III trial data showing a survival benefit are awaited, carboplatin is not yet standard-of-care for TNBC. The aim of this study was to examine the rate of pCR and the outcome for those treated with carboplatin and to examine the predictors of response to therapy.

**Methods:**

The retrospective series comprised 333 consecutive patients with TNBC (median follow-up time, 43 months). Adjuvant chemotherapy was given to 51% (*n* = 168) of patients and 29% (*n* = 97) received anthracycline–taxane NACT with carboplatin given to 9% (*n* = 31) of patients.

**Results:**

Overall, 25% (*n* = 78) of patients experienced a breast cancer recurrence and 22% (*n* = 68) died from disease. A pCR breast and pCR breast/axilla was more common in those who received carboplatin (*n* = 18, 58% and *n* = 17, 55%, respectively) compared those who did not (*n* = 23, 36% and *n* = 18, 28%, respectively) (*p* = 0.041 and *p* = 0.011, respectively). By multivariable analysis, carboplatin and high tumor grade were independent predictors of pCR breast/axilla (OR_non-pCR_ = 0.17; 95% CI 0.06–0.54; *p* = 0.002; and OR_non-pCR_ = 0.05, 95% CI 0.01–0.27; *p* < 0.001, respectively). pCR breast/axilla was an independent predictor of DFS (HR_non-pCR_=6.23; 95% CI 1.36–28.50; *p* = 0.018), metastasis-free survival (HR_non-pCR_ = 5.08; 95% CI 1.09–23.65; *p* = 0.038) and BCSS (HR_non-pCR_ = 8.52; 95% CI 1.09–66.64; *p* = 0.041).

**Conclusion:**

Carboplatin therapy and high tumor grade are associated with a significant increase in the rate of pCR, which is an independent predictor of outcome. These data support the use of carboplatin in NACT for TNBC, while results from phase III studies are awaited.

**Electronic supplementary material:**

The online version of this article (10.1007/s10549-018-5066-6) contains supplementary material, which is available to authorized users.

## Background

Triple negative breast cancer (TNBC) is an operational term used to define tumors that lack expression of estrogen receptor (ER), progesterone receptor (PR) and lack amplification or overexpression of human epidermal growth factor receptor 2 (HER2) [1, 2]. Approximately 20% of breast cancers are TNBC [3]. A small proportion of TNBCs, including adenoid cystic, secretory, adenosquamous and carcinoma, have low aggressive potential and follow an indolent course. However, the majority of TNBCs, most of which are invasive ductal carcinomas, are associated with high rates of early distant recurrence and short survival times compared to other breast cancer subtypes [1, 2, 4–9]. Almost all women with metastatic TNBC will die of their disease within 5 years of diagnosis [1, 3, 10, 11]; however, those who remain disease-free 8 years after initial diagnosis rarely die of breast cancer [1, 2, 4], unlike other breast cancer subtypes.

A subgroup of TNBCs are inherently chemosensitive and achieve a pathological complete response (pCR) following combination anthracycline/taxane-based neo-adjuvant chemotherapy (NACT) [2, 12–16]. These patients have a higher rate of survival compared to those with residual disease [2, 17, 18]. The basis for this variation in response to chemotherapy and survival remains unclear. TNBCs show considerable molecular heterogeneity, and no predictive markers have been identified to date [16, 17, 19–21]. Consequently, efforts have focused on modifying the standard chemotherapeutic regimen using new therapeutic agents to improve outcome.

The observation that many TNBCs exhibit dysfunction of DNA damage repair pathways [22, 23] has led to the inclusion of platinum salts in the treatment regimen with improved rates of pCR reported both in *BRCA* mutation carriers and in sporadic tumors in several phase II trials [12, 13, 24–29]. These data are encouraging; however, platinum salts are not yet part of the standard treatment for TNBC and a definite survival benefit remains to be demonstrated [30, 31]. Despite this, carboplatin was added to the NACT regimen for TNBC in our institution in 2013 based on phase II trial data. The aim in this study was to examine the effect of carboplatin on outcome for patients with TNBC and to identify biomarkers predictive of response to treatment and outcome. Outcome data from a retrospective series of 333 patients diagnosed with TNBC, including patients treated with carboplatin added to anthracycline–taxane NACT, are presented. This cohort represents one of the largest clinical cohorts of TNBC in the literature, reported outside of the clinical trial setting.

## Materials and methods

### Patient series

The series comprised 333 consecutive patients who were diagnosed with TNBC at Galway University Hospital between January 1, 2000, and December 31, 2015. Cases were ER and PR negative by immunohistochemistry (IHC) [32] and HER2 negative by IHC and/or fluorescent in situ hybridization (FISH). Twenty-seven cases with low ER expression (Allred score 3) [32] were included. Tumor type and grade were re-evaluated [33, 34] on full-face sections of the resections or of the pre-treatment biopsy in cases where a pCR was achieved [GC, AS]. An adverse event was defined as a local ipsilateral recurrence; a contralateral breast cancer; a distant event [35], and each was recorded as TNBC or non-TNBC. A non-breast cancer in any organ outside the breast, excluding non-melanoma skin cancer, was recorded. Deaths from breast cancer were recorded, and non-cancer-related deaths were excluded.

Adjuvant chemotherapy was the standard-of-care for TNBC prior to 2010. This changed to NACT with an anthracycline–taxane combination after 2010 with carboplatin incorporated into the NACT regime from 2013. Four patients who had very low volume oligometastatic disease in distant sites shortly after diagnosis were treated with NACT and were included with this group for analysis based on the intention-to-treat. Sixty-eight patients did not receive chemotherapy for different reasons including metastatic disease diagnosed at (*n* = 13) or shortly after diagnosis (*n* = 2); patient declined or was unfit for treatment (*n* = 27) or for reasons that were not specified (*n* = 26). Patients who had metastasis at diagnosis were excluded from analyses of DFS and MFS.

Tumors were staged according to guidelines [36]. In accordance with recommendations for standardized reporting of pCR [15, 37], a pCR was defined as no residual invasive disease in the breast, with or without residual in situ disease, and no residual disease in the axillary lymph nodes (ypT0/isypN0) [36] and is referred to as pCR breast/axilla. For the purposes of statistical analysis, pCR breast (ypT0/is) also was also examined as an endpoint.

### Immunohistochemistry

TNBC status was confirmed by IHC using a tissue microarray (TMA) containing 2 mm tumor cores in cases that had sufficient tumor using ER (SP1 Rabbit Monoclonal antibody; Thermo-Scientific; Dilution 1:00); PR (16/SAN27 Mouse Monoclonal antibody; Lecia; Dilution 1:200); and HER2 (4B5 Rabbit Monoclonal antibody; Roche; pre-filled dilution) with HER2 FISH as required and reported according to current guidelines [38, 39]. Cases were classified as basal if there was any positivity for cytokeratin (CK) 5/6 (D5/16, B4 Mouse Monoclonal antibody; Dako; Dilution 1:100) or epidermal growth factor receptor (EGFR) (EGFR.25; Leica; Dilution 1:100) [4] on full-face sections at diagnosis for some cases; on core biopsies for cases that had a pCR post-NACT; and on TMA sections for other cases.

### Statistical analysis

The Kruskal Wallis test with Dunn’s post-estimation and Fisher’s exact test were used to examine the differences in age and clinico-pathological characteristics between treatment groups, respectively. Differences in follow-up and survival times were analyzed using Oneway Anova with Bonferroni post-estimation. Associations between clinico-pathological characteristics and survival were assessed using log-rank tests with two proportion tests to estimate the effect (difference in proportions) of significant variables. Pearson *χ*^2^ testing was used to assess the interaction between variables. Both univariate and multivariate Cox regression analyses were used to calculate hazard ratios (HR) and 95% confidence intervals (95% CI), adjusting for variables. Logistic regression analysis was used to calculate odds ratio (OR) and 95% CI to adjust for prognostic variables. Test for trend of survivor testing assessed the impact of increasing prognostic variables on outcomes. Kaplan–Meier estimates were plotted for disease-free survival (DFS), metastases-free survival (MFS) and breast cancer-specific survival (BCSS). *p values* reported were two-tailed, and *p* < 0.05 was considered statistically significant. Statistical analysis was performed using Stata/IC (v14.0) and SPSS (v24).

## Results

### Patient and tumor characteristics

Patient demographics and tumor characteristic are shown in Table 1. Overall, 51% of patients received adjuvant chemotherapy (*n* = 168) and 29% (*n* = 97) patients received NACT. Patients who received NACT were younger than those in the other treatment groups (*p* < 0.001). Tumor type differed between the groups (*p* = 0.004). Lobular carcinomas accounted for 5% of TNBCs, most of which were pleomorphic ILC, and were more common in those who did not receive chemotherapy. More of those who received NACT had grade 2 tumors compared to patients treated with adjuvant therapy (*p* < 0.001). The follow-up time was shorter for those who received NACT compared to those who received adjuvant therapy (median 30 months; range 5–126 and median 64 months, range 2–186, respectively), which paralleled the shift from adjuvant chemotherapy to NACT since 2010.

**Table 1:** Patient and tumor characteristics

Parameter		All patients	NACT	Adjuvant CT	No CT	*p* Value	*p* Value
		*n* (%)	*n* (%)	*n* (%)	*n* (%)	None versus NACT versus Adjuvant	NACT versus Adjuvant CT
Number of patients		333	97 (29%)	168 (51%)	68 (20%)		
Age at diagnosis (years)	Median	55	48	55	75	< 0.001	< 0.001
	Range	24–92	24–73	30–79	30–92		
Menopausal	Pre	119 (36%)	57 (59%)	58 (35%)	4 (6%)	< 0.001	< 0.001
Status	Post	200 (60%)	33 (34%)	105 (62%)	62 (91%)		
	Unknown	14 (4%)	7 (7%)	5 (3%)	2 (3%)		
BRCA mutation	None	240 (72%)	69 (71%)	129 (77%)	42 (62%)	0.086	0.807
	BRCA1 mutation	12 (4%)	4 (4%)	8 (5%)	0		
	BRCA2 mutation	7 (2%)	2 (2%)	5 (3%)	0		
	Unknown	74 (22%)	22 (23%)	26 (15%)	26 (38%)		
Family history of breast cancer	No	123 (55%)	40 (56%)	66 (54%)	17 (55%)	0.956	0.763
	Yes	101 (45%)	31 (44%)	56 (46%)	14 (45%)		
Tumor type	Ductal	271 (81%)	87 (90%)	138 (82%)	46 (68%)	0.004	0.595
	Lobular	16 (5%)	1 (1%)	7 (4%)	8 (15%)		
	Metaplastic	15 (5%)	4 (4%)	6 (4%)	5 (7%)		
	Medullary	14 (4%)	4 (4%)	8 (5%)	2 (3%)		
	Apocrine	9 (3%)	1 (1%)	2 (1%)	6 (9%)		
	Other^a^	4 (1%)	0	3 (2%)	1 (1%)		
	Unknown	4 (1%)	0	4 (2%)	0		
Tumor grade	1	1 (0.5%)	0	1 (1%)	0	< 0.001	< 0.001
	2	63 (19%)	27 (28%)	15 (9%)	21 (31%)		
	3	265 (79.5%)	70 (72%)	148 (88%)	47 (69%)		
	Unknown	4 (1%)	0	4 (2%)	0		
Basal status^b^	Positive	254 (76%)	79 (81%)	131 (78%)	44 (65%)	0.225	0.227
	Negative	57 (17%)	12 (12%)	31 (18%)	14 (20%)		
	Unclassified	22 (7%)	6 (6%)	6 (4%)	10 (15%)		
pT stage	1	78 (33%)	NA	57 (34%)	21 (34%)	0.088	NA
	2	121 (51%)		92 (55%)	29 (48%)		
	3	11 (5%)		8 (4.5%)	3 (5%)		
	4	14 (6%)		6 (4%)	8 (13%)		
	Unknown	12 (5%)		5 (3%)	7		
pN stage	0	140 (59%)	NA	104 (62%)	36 (53%)	0.619	NA
	1	43 (18%)		33 (20%)	10 (15%)		
	2	19 (8%)		12 (7%)	7 (10%)		
	3	14 (6%)		8 (5%)	4 (6%)		
	Unknown	22 (9%)		11 (6%)	11 (16%)		
ypT stage	0	35 (36%)	35 (36%)	NA	NA	NA	NA
	In situ	8 (8%)	8 (8%)				
	1	27 (28%)	27 (28%)				
	2	12 (13%)	12 (13%)				
	3	5 (5%)	5 (5%)				
	4	10 (10%)	10 (10%)				
ypN stage	0	64 (66%)	64 (66%)	NA	NA	NA	NA
	1	11 (11%)	11 (11%)				
	2	13 (14%)	13 (14%)				
	3	8 (8%)	8 (8%)				
	Unknown	1 (1%)	1(1%)				
M stage	0	316 (95%)	93 (96%)	168 (100%)	55 (81%)	< 0.001	0.008
	1	17 (5%)	4 (4%)	0	13 (19%)		
Chemotherapy	Anthra., Taxane	124 (47%)	64 (66%)	60 (36%)	NA	NA	NA
Agents^c^	Taxane	64 (24%)	0	64 (38%)			
	Anthra., Taxane, carboplatin	31 (12%)	31 (32%)	0			
	Anthra	30 (11%)	0	30 (18%)			
	Other	6 (2%)	0	6 (3%)			
	Unknown regimen	10 (4%)	2 (2%)	8 (5%)			
Outcome	All new adverse events^d,e^	88 (28%)	21 (23%)	46 (27%)	21 (38%)	0.121	0.395
	Ipsilateral recurrence (TNBC)	13 (4%)	1 (1%)	8 (5%)	4 (7%)		
	Ipsilateral recurrence (non-TNBC)	2 (0.6%)	0	1 (0.6%)	1 (2%)		
	Contralateral IBC (TNBC)	3 (1%)	0	3 (2%)	0		
	Contralateral IBC (non-TNBC)	1 (0.3%)	1 (1%)	0	0		
	Distant event	59 (19%)	19 (20%)	26 (15%)	14 (25%)		
	Other cancer (non-breast)	10 (3%)	0	8 (5%)	2 (4%)		
	Death from disease^f^	68 (22%)	18 (19%)	25 (16%)	25 (39%)		
Follow-up time (months)	Median	43	30	64	24	< 0.001	< 0.001
	Range	0–199	5–126	2–186	0–199		

*Anthra* Anthracycline, *CT* chemotherapy, *n* number of patients, *IBC* invasive breast cancer, *NACT* neoadjuvant chemotherapy, *NA* not applicable, *TNBC* triple negative breast cancer

^a^Other tumor types include micropapillary (*n* = 1); mixed ductal-lobular (*n* = 1), mixed ductal-micropapillary (*n* = 1) and papillary carcinoma (*n* = 1); ILC comprised pleomorphic ILC (*n* = 13) and classic ILC (*n* = 3)

^b^Basal status: any positivity for either cytokeratin 5/6 or EGFR by immunohistochemistry

^c^Chemotherapeutic agents listed for patients known to have received either adjuvant chemotherapy or NACT

^d^All new adverse events were classified according to Maastricht Delphi consensus criteria [35]. All distant events of breast cancer were TNBC. There were no adverse events that were either regional axillary or in situ disease. Non-melanoma skin cancer was excluded from other cancer that was non-breast. Metastatic disease at diagnosis was not regarded as a new event

^e^Number of patients evaluable for new adverse events excluding death from disease (DFS, MFS) = 316: NACT (*n* = 93), adjuvant chemotherapy (*n* = 168), no CT (*n* = 55)

^f^Number of patients evaluable for death (BCSS) = 321: NACT (*n* = 96), adjuvant therapy (*n* = 161), no chemotherapy (*n* = 64)

### Treatment

NACT consisted of an anthracycline–taxane combination with platinum included for 31 patients. Paclitaxel was given weekly for 12 weeks with carboplatin administered at an AUC = 5 mg*min/mL every 3 weeks, followed by dose dense doxorubicin plus cyclophosphamide. NACT was reasonably well tolerated, albeit with more toxicities recorded among those who received carboplatin: 19% (*n* = 6) versus 13% (*n* = 8) had a treatment delay; 19% (*n* = 6) versus 5% (*n* = 3) failed to complete their recommended course of treatment; and 7% (*n* = 2) versus 0% had a dose reduction in the platinum and non-platinum treated group, respectively. Despite failing to complete their recommended NACT, four of six patients treated with carboplatin achieved a pCR, whereas none of the three patients treated with non-platinum NACT who failed to complete their NACT achieved a pCR. In the adjuvant setting, most patients received a taxane; an anthracycline–taxane; or an anthracycline combination (Table 1). All adjuvant chemotherapy was well tolerated. Treatment delays were seen in 22%, 20% and 3% among anthracycline, anthracycline–taxane and taxane combinations, respectively, and dose reductions were very infrequent (< 5%). Adjuvant endocrine therapy was given in the case of cancers with low (< 10%) ER or PR expression, or for an ER positive recurrence.

### Outcomes

**Fig. 1:**
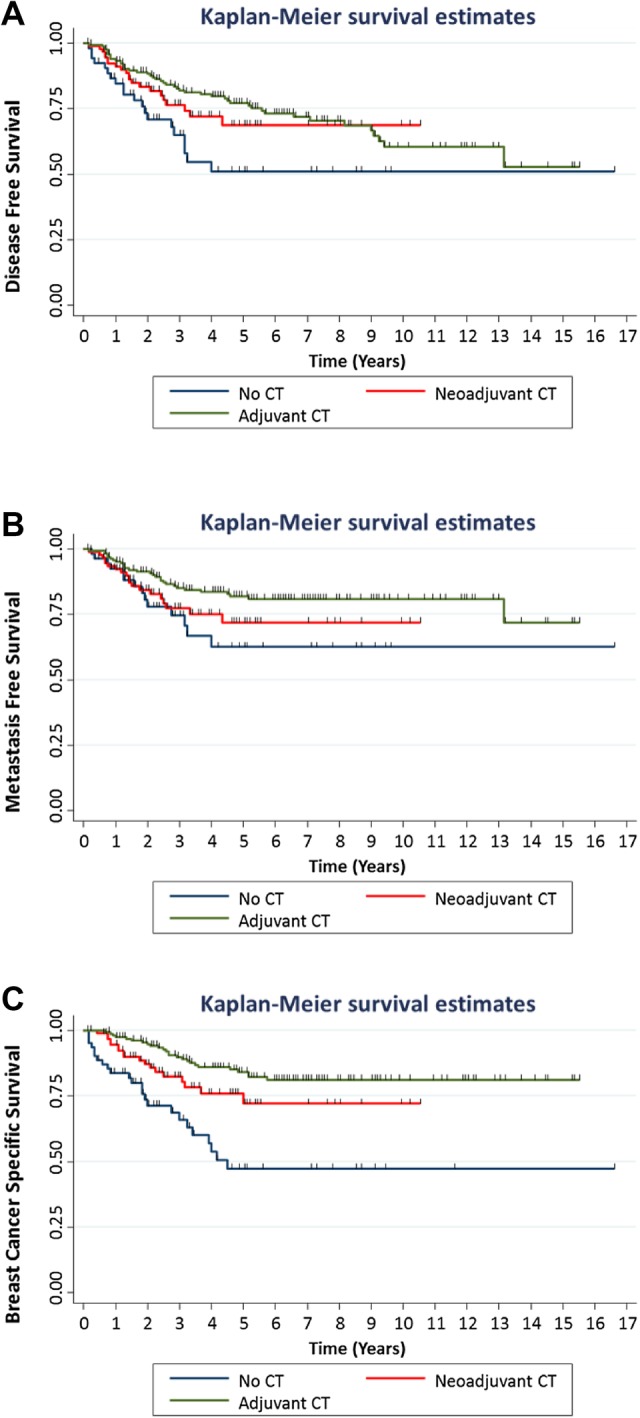
Disease-free, metastases-free and breast cancer-specific survival for patients according to schedule of chemotherapy. Kaplan–Meier cumulative survival curves for **a** disease-free survival and **b** metastases-free survival for patients who received adjuvant chemotherapy (CT) (*n* = 168), neoadjuvant CT (*n* = 93) or no CT (*n* = 55) (log-rank test DFS, *p* value = 0.024) and (log-rank test MFS, *p* value = 0.045). **c** Kaplan–Meier cumulative survival curves for breast cancer-specific survival for patients for patients who received adjuvant therapy (*n* = 161), neoadjuvant CT (*n* = 96) or no CT (*n* = 64) (log-rank test, *p* value < 0.001)

The median DFS, MFS and BCSS for all patients was 39.5, 40.5 and 44 months (range 0–199 months), respectively. The DFS, MFS and BCSS in the different treatment groups was compared (Fig. 1). There were statistically significant differences between the three treatment groups but there was no significant difference in DFS (log-rank test, *p* = 0.352) or MFS (log-rank test, *p* = 0.094) between patients receiving NACT versus adjuvant therapy. However, a marginally significant improvement in BCSS was seen in patients receiving adjuvant therapy compared to NACT (log-rank test, *p* = 0.049).

The number of new adverse events in the different treatment groups was similar (*p* = 0.121) (Table 1), and most of these (67%, *n* = 59) were distant recurrences of TNBC. Distant metastases were more common than local recurrences as a first event in the NACT group compared to the adjuvant therapy group (*p* = 0.029). Irrespective of whether local recurrence or distant metastatic disease developed first, there was no difference in the number of patients who ultimately developed metastatic disease in the NACT or adjuvant therapy groups (*p* = 0.527). A new non-TNBC breast cancer was uncommon (3%, *n* = 3); as was a cancer outside the breast (11%, *n* = 10), most of which occurred in those who received adjuvant therapy, possibly due to their longer follow-up time. Overall, 22% (*n* = 68) of patients died from TNBC; this included 64 patients who had non-metastatic disease at diagnosis. The time from recurrence to death was short for all patients (median 8 months; 0–33). There were no treatment-related deaths.

### Response to NACT

A pCR breast (ypT0/is) was observed in 43 patients (44%), including eight who had residual in situ disease only; 37 patients (38%) had a pCR breast/axilla (ypT0/isN0). A pCR breast was significantly more common in patients who received carboplatin (*n* = 18, 58%) compared to those who did not (*n* = 23, 36%) (Pearson *χ*^2^ = 4.81; *p* = 0.041). Likewise, a pCR breast/axilla was more frequent with carboplatin therapy (*n* = 17, 55%) than without it (*n* = 18, 28%) (Pearson *χ*^2^: 6.41, *p* = 0.011). Univariate logistic regression analysis (Table 2) revealed that the likelihood of both a pCR breast and pCR breast/axilla was highest after carboplatin (OR_non-pCR_ = 0.38; 95% CI 0.16–0.91, *p* = 0.030; OR 0.32; 95% CI 0.13–0.79; *p* = 0.013, respectively); and in grade 3 tumors (OR 0.15; 95% CI 0.05–0.47; *p* = 0.001; OR 0.08; 95% CI 0.02–0.36; *p* = 0.001, respectively). A family history of breast cancer increased the likelihood of a pCR breast (OR 0.34; 95% CI 0.13–0.90; *p* = 0.030) but not of a pCR breast/axilla (OR 0.51; 95% CI 0.19–1.31; *p* = 0.162); 61% of those with a family history of breast cancer achieved a pCR breast compared to 35% of those without a family history (Pearson *χ*^2^ = 4.85; *p* = 0.028). *BRCA1*/*2* mutation status did not increase the likelihood of a pCR, but four of the six *BRCA1*/*2* mutation carriers had a pCR breast, and the remaining two patients had a near total response (ypT1mi or ypT1b). None of the other parameters were associated with pCR on univariate analysis.

**Table 2:** Univariate and multivariable analyses of predictors of pCR breast and pCR breast/axilla

	*N*	OR^a^	95% CI	*p* Value
Univariate analysis				
pCR breast				
Age at diagnosis	97	1.02	0.98–1.05	0.434
Menopausal status	90	1.06	0.45–2.52	0.895
Family history of breast cancer	71	0.34	0.13–0.90	0.030
Tumor type	97	0.91	0.65–1.26	0.557
Tumor grade	97	0.15	0.05–0.47	0.001
Basal status^b^	91	0.63	0.18–2.26	0.477
Platinum-based NACT	95	0.38	0.16–0.91	0.030
pCR breast/axilla				
Age at diagnosis	97	1.01	0.98–1.05	0.454
Menopausal status	90	1.1	0.45–2.67	0.833
Family history of breast cancer	71	0.51	0.19–1.32	0.162
Tumor grade	97	0.08	0.02–0.36	0.001
Tumor type	97	1.04	0.73–1.48	0.815
Basal status^b^	91	0.49	0.12–1.95	0.311
Platinum-based NACT	95	0.32	0.13–0.79	0.013
Multivariable analysis				
pCR breast	95			
Age at diagnosis		1.00	0.96–1.05	0.892
Tumor grade		0.10	0.03–0.37	0.001
Platinum-based NACT		0.26	0.09–0.74	0.011
pCR breast/axilla	95			
Age at diagnosis		1.01	0.97–1.05	0.704
Tumor grade		0.05	0.01–0.27	< 0.001
Platinum-based NACT		0.17	0.06–0.54	0.002

*n* Number of patients, *OR* odds ratio, *CI* confidence interval, *NACT* neoadjuvant chemotherapy, *pCR* pathological complete response

^a^OR for a non-pCR by Logistic regression analysis

^b^Basal status: any positivity for either cytokeratin 5/6 or EGFR by immunohistochemistry

By multivariable analysis (Table 2), carboplatin therapy (OR 0.26; 95% CI 0.09–0.74; *p* = 0.011) and grade 3 histology (OR 0.10; 95% CI 0.03–0.37; *p* = 0.001) remained independently associated with a pCR breast and with a pCR breast/axilla (OR 0.17; 95% CI 0.06–0.54; *p* = 0.002, and OR 0.05; 95% CI 0.01–0.27; *p* < 0.001 for carboplatin and grade, respectively).

### DFS, MFS and BCSS

#### Patients treated with NACT

**Fig. 2:**
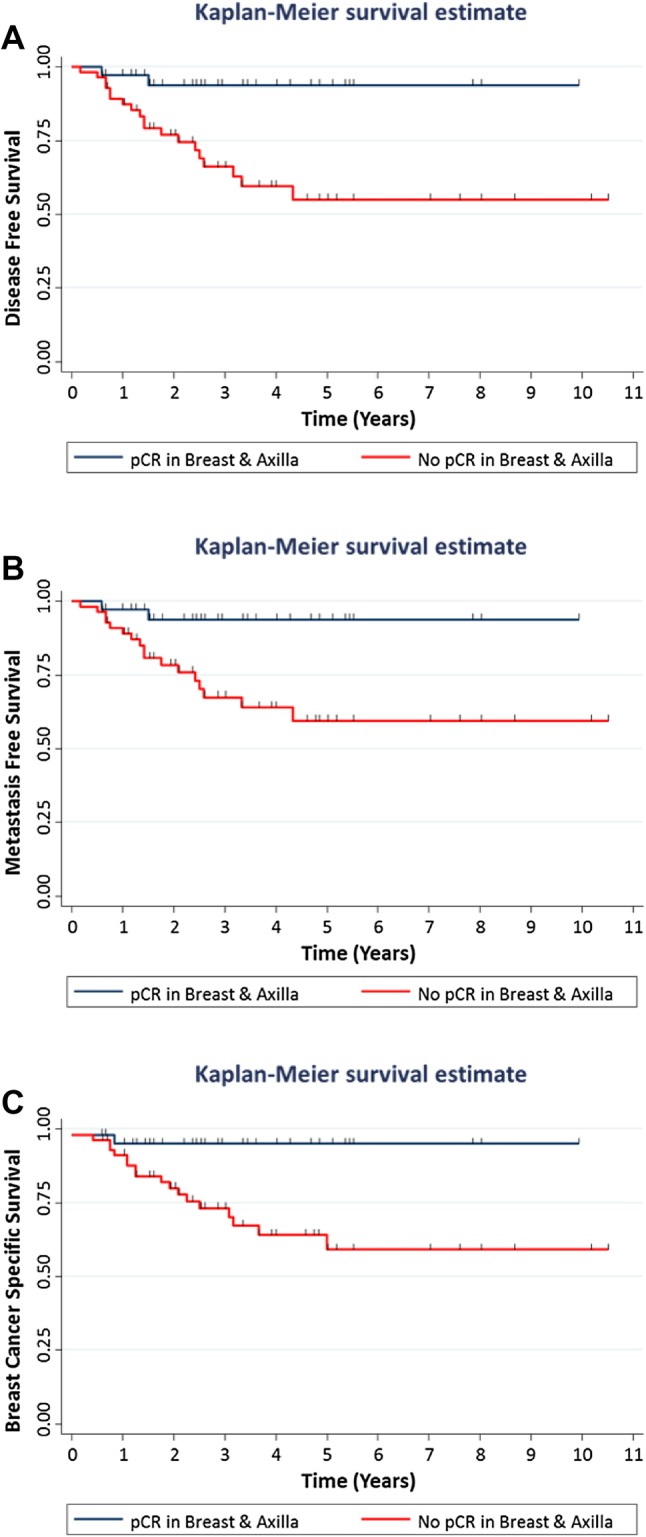
Disease-free, metastases-free and breast cancer-specific survival according to pathological response to NACT. Kaplan–Meier cumulative survival curves show the association between pathological complete response (pCR) breast/axilla and **a** disease-free survival (*n* = 93) log-rank test, *p* value = 0.003; **b** metastases-free (*n* = 93) log-rank test, *p* value = 0.007; and **c** breast cancer-specific survival (*n* = 96) log-rank test, *p* value = 0.005. A pCR breast/axilla is defined as ypT0/isN0

By univariate analyses (Supplementary Tables 1 and 2), both pCR endpoints increased the likelihood of an improved DFS, MFS and BCSS. The magnitude of the effect of pCR on outcome was greatest for pCR breast/axilla for DFS (HR = 6.66; 95% CI 1.54–28.58; *p* = 0.011); for MFS (HR = 5.90; 95% CI 1.36–25.56; *p* = 0.018); and for BCSS (HR = 10.30; 95% CI 1.37–77.38; *p* = 0.023) (Fig. 2).

There was a significant trend of association between increasing ypT category and adverse DFS (*χ*^2^ test for trend, 13.56; *p* = 0.002), MFS (*χ*^2^ test for trend, 12.90; *p* = 0.0003) and BCSS (*χ*^2^ test for trend, 11.14; *p* = 0.0008). However, there was no significant difference in outcome for patients with ypT1 versus ypT0/ypT0/is disease. A significant trend of association was also observed between an increasing number of positive nodes and adverse DFS (*χ*^2^ test for trend, 18.32; *p* < 0.0001), MFS (*χ*^2^ test for trend, 17.20; *p* < 0.001) and BCSS (*χ*^2^ test for trend, 33.56; *p* < 0.001). The outcome was worst for those with ypN3 disease (Supplementary Tables 1 and 2).

On multivariable analyses (Table 3), a pCR breast/axilla was the only independent predictor of DFS (HR = 6.23; 95% CI 1.36–28.50; *p* = 0.018), MFS (HR = 5.08; 95% CI 1.09–23.65; *p* = 0.038) and BCSS (HR = 8.52; 95% CI 1.09–66.64; *p* = 0.041), when adjusted for age at diagnosis, tumor grade, tumor type, basal status and the administration of carboplatin. In a separate multivariable model using the same co-variables, pCR breast also remained the only independent predictor of DFS, MFS and BCSS (Supplementary Table 3).

**Table 3:** Multivariable analysis of DFS, MFS and BCSS for patients treated with NACT

	*n*	HR^a^	95% CI	*p* Value
Disease-free survival	85			
Age at diagnosis		0.99	0.95–1.04	0.776
Tumor grade		1.05	0.39–2.81	0.926
Tumor type		0.64	0.27–1.49	0.298
Basal status^b^		1.61	0.36–7.20	0.530
Platinum-based therapy		0.68	0.18–2.56	0.566
pCR breast/axilla^c^		6.23	1.36–28.50	0.018
Metastasis-free survival	85			
Age at diagnosis		0.99	0.95–1.04	0.841
Tumor grade		0.85	0.31–2.37	0.759
Tumor type		0.67	0.30–1.52	0.343
Basal status^b^		1.47	0.32–6.63	0.619
Platinum-based therapy		0.69	0.18–2.65	0.590
pCR breast/axilla^c^		5.08	1.09–23.65	0.038
Breast cancer-specific survival	88			
Age at diagnosis		1.01	0.96–1.06	0.714
Tumor grade		1.14	0.40–3.26	0.813
Tumor type		0.77	0.40–1.49	0.441
Basal status^b^		1.32	0.29–6.07	0.719
Platinum-based therapy		0.310	0.04–2.50	0.272
pCR breast/axilla^c^		8.52	1.09–66.64	0.041

*n* Number of patients, *HR* hazard ratio, *CI* confidence interval

^a^Cox regression survival analysis

^b^Basal status: any positivity for either cytokeratin 5/6 or EGFR by immunohistochemistry

^c^HR for non-pCR using pCR as the baseline value

**Fig. 3:**
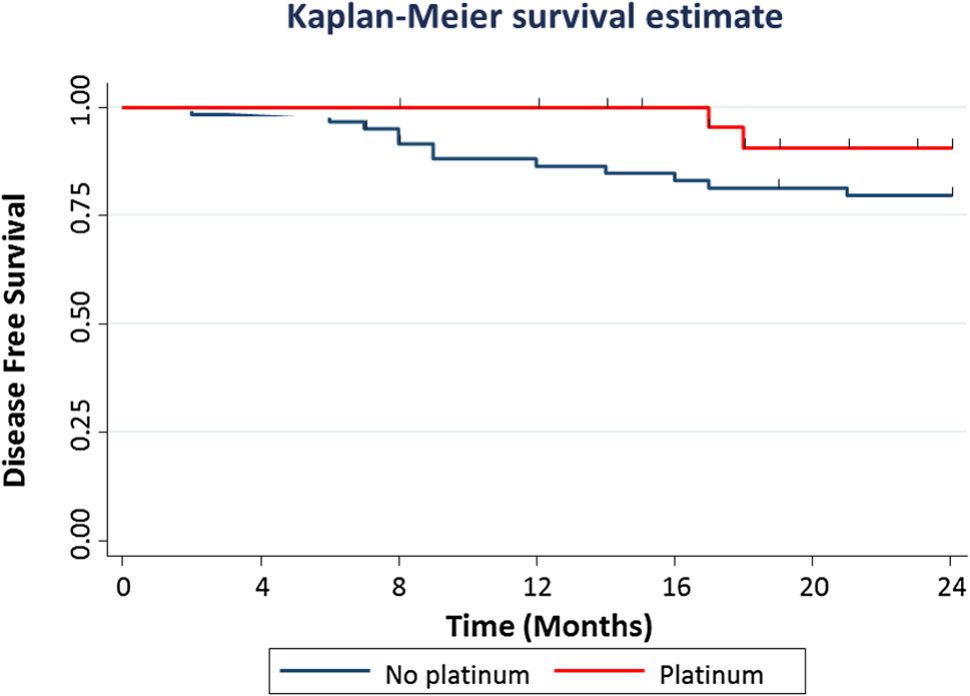
Disease-free survival for patients who received NACT according to the administration of platinum agents. Kaplan–Meier curves show the disease-free survival for patients stratified according to the administration of platinum-based NACT. The analysis was confined to 24-month follow-up period because the follow-up time for patients who received a platinum agent was short. A new event was observed in two of 31 patients who received a platinum and in 12 of 60 patients who received standard anthracycline–taxane-based NACT (log-rank test, *p* value = 0.262)

The association between carboplatin therapy and outcome limited to 24-month follow-up time was examined (Fig. 3) because the median follow-up time for patients who received carboplatin was short (18 months; range 8–34). DFS was improved with platinum therapy but the association did not reach statistical significance on univariate (HR_non-pCR_ = 0.34; 95% CI 0.75–1.50; *p* = 0.153) or multivariable analysis (Supplementary Table 4).

#### Patients who did not receive NACT

Univariate analysis (Supplementary Tables 1 and 2) revealed that increasing tumor size (pT) and nodal status were a significant risk factor for adverse DFS (*χ*^2^ test trend 8.83, *p* = 0.003; and 29.06, *p* < 0.001, respectively); MFS (*χ*^2^ test trend 24.92, *p* < 0.001) and BCSS (*χ*^2^ test trend 32.40, *p* < 0.001 and 29.46, *p* < 0.001, respectively). A strong positive correlation between tumor size and nodal status (Pearson *χ*^2^ = 73.01, *p* < 0.001) was noted. However, there was no difference in outcome for pT2 and pT1 tumors or between pN1 and node negative disease.

Multivariable analysis (Table 4) revealed that only nodal stage at diagnosis was an independent predictor of DFS (HR = 1.75; 95% CI 1.30–2.34; *p* < 0.001), and both nodal status (HR = 1.58; 95% CI 1.13–2.23; *p* = 0.008) and tumor size (HR = 1.68; 95% CI 1.10–2.58; *p* = 0.018) were independent predictors of MFS. When those with M1 at diagnosis were excluded, pT was the only independent predictor of BCSS (HR = 2.08; 95% CI 1.39–3.11; *p* < 0.001). In a separate analysis including those with M1 disease at diagnosis, metastasis and tumor size were independent predictors of BCSS.

**Table 4:** Multivariable analysis of DFS, MFS and BCSS in patients treated with adjuvant or no chemotherapy

	*n*	HR^a^	95% CI	*p* Value
Disease-free survival	206			
Age at diagnosis		1.02	0.99–1.04	0.128
Tumor grade		0.78	0.40–1.51	0.455
pT		1.07	0.73–1.59	0.723
pN		1.75	1.30–2.34	< 0.001
Metastases-free survival	206			
Age at diagnosis		1.01	0.99–1.04	0.275
Tumor grade		1.18	0.47–3.04	0.340
pT		1.70	1.10–2.62	0.017
pN		1.57	1.11–2.21	0.009
Breast cancer-specific survival	203			
Age at diagnosis		1.01	0.99–1.04	0.238
Tumor grade		1.46	0.57–3.76	0.427
pT		2.05	1.37–3.10	0.001
pN		1.30	0.91–1.84	0.154
M1 at diagnosis		7.66	2.72–20.08	< 0.001

*n* number of patients, *HR* hazard ratio, *CI* confidence interval, *nc* not calculable, *M1* metastatic disease

^a^HR for non-pCR by Cox regression survival analysis using pCR as the baseline value

There was no association between tumor type and outcome (Table 3, Supplementary Tables 1, 2). Metaplastic carcinomas were uncommon (*n* = 15); however, we observed that only five of the 15 patients died from disease after a median time to recurrence of 26 months (range 0–44 months) and median time to death of 32 months (range 2–58 months). There was no difference in survival for patients with metaplastic carcinoma between the three treatment groups.

## Discussion

We show in a retrospective series that patients with TNBC who received carboplatin with anthracycline–taxane-based NACT have significantly higher rate of pCR compared to those who received anthracycline/taxane alone. A pCR breast and a pCR breast/axilla, achieved in 58% and 55% of those treated with carboplatin, respectively, were the only independent predictors of survival, although follow-up time was short. In patients who did not received NACT, only traditional parameters of nodal status and tumor size had prognostic significance.

Several studies have shown the favorable association between pCR and outcome [40–43]; patients with TNBC who attain a pCR have an outcome that is comparable to that for patients with non-TNBC who attain a pCR [2, 14, 15, 44]. It is generally accepted that a pCR breast/axilla is a stronger predictor of outcome than a pCR breast, which was shown in our series where a pCR breast/axilla was a stronger predictor of BCSS than a pCR breast for analyses of BCSS. However, this difference in benefit was not observed for DFS or MFS on multivariate analysis, where both pCR endpoints improved DFS and MFS comparably. The benefit of a pCR in terms of survival shown in our data has been reported by others [13, 15, 45, 46]. However, the prognostic value of pCR has not yet been validated at trial level, as reported in the pooled analysis of almost 12,000 patients [15]; and it is likely that other variables affect the relationship between pCR and survival that should be examined in future trials examining the prognostic value of pCR [46, 47].

The rate of pCR in our series is comparable to that reported in two prospective randomized phase II clinical trials evaluating carboplatin with anthracycline–taxane NACT in stage II-III TNBC. In the GeparSixto trial, carboplatin and bevacizumab resulted in an increase from 37 to 53%, in the rate of pCR in 158 patients [13]. The Alliance study [12] reported a pCR of 54% in those treated with carboplatin with an alkylating agent. Similarly, in a smaller series, Ando et al. [26] reported a pCR in 61% for those treated with carboplatin with anthracycline–taxane. Only the earlier GEICAM randomized trial failed to show any improvement in pCR rate with carboplatin [48]. The phase II adaptively randomized I-SPY 2 study [25] which assessed the addition of a poly-ADP-ribose-polymerase (PARP) inhibitor, veliparib, plus carboplatin to anthracycline–taxane NACT also reported a pCR in 51% of those who received veliparib and carboplatin.

The data from these studies are promising; however, the optimal way of incorporating platinum agents in the NACT regimen is not yet established [49]. In the GeparSixto and Ca.Pa.Be studies, different anthracycline–taxane combinations were used and bevacizumab was given [13, 28]. The dose of carboplatin also differed between studies: GEICAM [48], CALGB 40603 [12] and I-SPY2 [25] used carboplatin every 3 weeks at an AUC = 6; Ando et al. [26] used carboplatin every 3 weeks at an AUC = 5; Ca.Pa.Be [28] used weekly carboplatin at AUC = 2 and the GeparSixto (13) used weekly carboplatin at an AUC = 1.5. In our institution, paclitaxel is given weekly for 12 weeks with carboplatin administered at an AUC = 5 every 3 weeks, followed by dose dense doxorubicin plus cyclophosphamide. Platinum agents are reportedly associated with increased toxicity relative to the standard chemotherapeutic regimen [12, 13]. Some suggest that carboplatin plus paclitaxel may have less hematological toxicities than carboplatin plus docetaxel [50–52] but others report good tolerance of carboplatin plus docetaxel in an anthracycline-free regimen [53]. In our cohort, toxicities and treatment delays were increased in those who received carboplatin, nonetheless most of those who received carboplatin but did not complete their still achieved a pCR.

Tumor grade was the only other independent predictor of pCR in this study and is in keeping with reported increased chemo-sensitivity for grade 3 compared to low-grade TNBCs [16]. This emphasizes the importance of accurate tumor grading in pre-NACT tumor material. Although most TNBCs are grade 3, we and others have recorded that between 16 and 21% of TNBCs are not high grade in series that exclude the low-grade indolent subtypes of TNBC [54, 55, present series]. Tumour histological type was not a prognostic factor in our series. The proportion of ILC in our series (5%) was greater than the 1% reported by others in 20,000 to 90,000 TNBCs [54–56]. However, it was comparable to the 7.7% of ILC recorded in a series 841 TNBCs [57], which suggests that the discrepancy may relate to the size of series. Metaplastic carcinomas were also uncommon, but we noted that two-thirds of patients with this subtype were disease-free after a median follow-up of 40 months (range 2–154 months). None of the other parameters examined in this study had prognostic or predictive significance.

Basal status by IHC was not informative, which contrasts with reports of adverse outcome for basal TNBCs [4] and with data showing higher rates of pCR for ‘Basal-like 1’ TNBCs defined by the TNBC type-4 [16, 58]. There is, as yet, no consensus on either the definition or on the clinical significance of the basal phenotype. Tumors identified as basal using different platforms overlap [4, 6, 16, 17, 19, 20, 59], but different platforms do not characterize the same tumors as basal. The majority of TNBCs in our series arose in the sporadic setting; however, a pCR breast was significantly associated with a family history of breast cancer, which may point to enhanced chemo-sensitivity or possibly ‘BRCA-ness’ in those with a family history [22, 23].

The overall outcome and pattern of recurrence for all patients in our series is in line with that reported by others [1, 2, 4–8]. Early distant metastases were more common that local recurrences as a first event, and the median survival time from first was only 8 months. In the non-neoadjuvant setting, tumor size and nodal status were the only predictors of outcome with a significant positive trend of association between these variables and outcome observed. However, there was no survival difference between patients with pT2 versus pT1 disease or between those with pN1 versus pN0 disease. This is at variance with other reports [5], in which outcome was related to nodal positivity versus node negative disease and not the number of positive nodes. This inconsistency may be explained by the small number of cases with N1 disease (*n* = 48) in our series. A nonlinear correlation between both nodal status and tumor size with outcome in TNBC has been observed by others [1, 60, 61] which suggests that tumor size and extent may be less valid for TNBC with its more aggressive biological behavior than for breast cancer as a whole.

There were limitations with this study. First, the median follow-up time for the NACT group was 30 months and only 18 months for those received carboplatin, reflecting the change in chemotherapy practice over time. Notwithstanding, the natural history of early recurrences peaking between within 3 years of diagnosis for TNBCs [1, 2, 4–8] mitigates to some degree the effect of short follow-up time on DFS. Also, the size of our series was not large enough to explore prognostic differences between subsets of TNBC. However, this study represents one of the largest clinical TNBC cohorts reported; we identified very few studies, performed outside of clinical trials, with larger cohorts of TNBC patients [5, 62–64]. These focused on surgical procedures [62, 63] or loco-regional recurrences as primary outcomes [64–66]. Even when clinical trials are included, this cohort represents a large, significant patient sample as only the BEATRICE (*n* = 2591) [65], GeparQuinto (*n* = 663) [66], NSABP-B40 (*n* = 479) [67] and CALGB 40603 (*n* = 433) [12] trials had larger TNBC patient cohorts.

The ypT stage assigned by the reporting breast pathologist at the time of diagnosis was used for analyses. Other systems that measure the degree of response to chemotherapy [68, 69] were reported inconsistently before 2013 and were not used for analysis. The Residual Cancer Burden score may be more promising than the TNM system for post-NACT staging; it provides an index that is predictive of long-term survival with reports of good reproducibility [37, 69–71]. Finally, 27 patients with low ER expression (1–9% positivity) were included. The optimal treatment for these ‘ER-poor’ cancers is not defined. In our institution, these are treated as ER negative disease, although some receive adjuvant endocrine therapy. Whether including these patients could skew our results is a legitimate question; however, molecular subtype analysis suggests that the majority of these cancers have the same profiles and clinical outcome as ER negative disease [72, 73].

In summary, our data demonstrate that carboplatin added to anthracycline–taxane NACT significantly improves the rate of pCR in TNBC and that pCR is the only independent predictor of outcome, albeit at relatively short follow-up time. Grade 3 histology was the only other independent predictor of pCR, underscoring the importance of accurate evaluation of grade in TNBCs pre-NACT. Platinum therapy is not yet standard-of-care for TNBC. While awaiting results from phase III trials examining the survival advantage for platinums (NCT02488967; NCT02445391), our results add to the data supporting the incorporation of carboplatin with anthracycline–taxane NACT [74]. It remains unclear whether all or a subset of TNBCs derive benefit from platinum and identification of predictive biomarkers of response are required.

## Electronic supplementary material

Below is the link to the electronic supplementary material.

Supplementary material 1 (DOCX 15 KB)

Supplementary material 2 (DOCX 16 KB)

Supplementary material 3 (DOCX 17 KB)

Supplementary material 4 (DOCX 15 KB)

## Data Availability

Data that support this study are available upon reasonable request.

## Abbreviations

AUC: Area under the curve
BCSS: Breast cancer-specific survival
BCT: Breast conserving therapy
*BRCA1*/*2*: Breast cancer gene 1/2
CALGB: Cancer and Leukemia Group B
CI: Confidence interval
CK: Cytokeratin
DFS: Disease-free survival
EGFR: Epidermal growth factor receptor
ER: Estrogen receptor
FISH: Fluorescent in situ hybridization
GUH: Galway University Hospital
HER2: Human epidermal growth factor receptor 2
H&E: Hematoxylin and eosin stain
HR: Hazard ratio
IHC: Immunohistochemistry
M1: Metastatic disease
MFS: Metastasis-free survival
NACT: Neoadjuvant chemotherapy
NSABP: National Surgical Adjuvant Breast and Bowel Project
NST: No special type
OR: Odds ratio
OS: Overall survival
PARP: Poly-ADP-ribose-polymerase
pCR: Pathological complete response
PR: Progesterone receptor
TMA: Tissue microarray
TNBC: Triple negative breast cancer
