# Supplementary material for: Outcome for triple negative breast cancer in a retrospective cohort with an emphasis on response to platinum-based neoadjuvant therapy
Source: Breast Cancer Res Treat. 2018 Nov 28;174(1):1–13. doi: 10.1007/s10549-018-5066-6 (PMC6418073; doi:10.1007/s10549-018-5066-6)
Supplement: Supplementary file 1 — Supplementary material 1 (DOCX 15 KB) [file 10549_2018_5066_MOESM1_ESM.docx]

**Supplementary Table 1 Univariate analysis of DFS and MFS for all patients**

| **Parameter** |  | **Disease Free Survival** | | | | **Metastasis Free Survival** | | | |
| --- | --- | --- | --- | --- | --- | --- | --- | --- | --- |
|  |  | **N** | **HR^a^** | **95% CI** | **p-value** | **n** | **HR^a^** | **95% CI** | **p-value** |
| **Age at Diagnosis** |  | 315 | 1.01 | 0.99-1.03 | 0.141 | 316 | 1.00 | 0.99-1.03 | 0.449 |
| **Menopausal Status** |  | 302 | 1.14 | 0.72-1.79 | 0.573 | 303 | 0.96 | 0.57-1.62 | 0.878 |
| **Tumour Grade (2 vs. 3)** |  | 310 | 0.92 | 0.53-1.59 | 0.769 | 311 | 0.78 | 0.42-1.45 | 0.435 |
| **Tumour Type** |  | 277 | 1.02 | 0.88-1.18 | 0.825 | 278 | 0.96 | 0.79-1.18 | 0.710 |
| **Basal Status^b^** |  | 299 | 0.96 | 0.56-1.64 | 0.886 | 299 | 0.94 | 0.50-1.78 | 0.855 |
| **pT Stage^c^** | **pT1**  **pT2**  **pT3**  **pT4** | 215 | 1.0  0.87  1.93  5.23 | 0.49-1.51  0.77-4.79  2.11-12.99 | 0.612  0.159  <0.001 | 215 | 1.0  1.43  3.45  11.43 | 0.67-3.04  1.08-11.02  4.13-31.60 | 0.352  0.036  <0.001 |
| **pN Stage^c^** | **pN0**  **pN1**  **pN2**  **pN3** | 207 | 1.0  0.60  3.22  6.43 | 0.26-1.38  1.58-6.56  3.04-13.57 | 0.230  0.001  <0.001 | 207 | 1.0  1.04  4.94  6.35 | 0.42-2.61  2.22-11.01  2.52-16.04 | 0.939  <0.001  <0.001 |
| **ypT Stage^d^** | **ypT0**  **ypTis**  **ypT1**  **ypT2**  **ypT3 ypT4** | 93 | 1.0  nc  2.54  4.44  5.40  7.84 | 0.63-10.15  1.06-18.6  0.9-32.37  1.87-32.85 | 0.188  0.041  0.065  0.005 | 93 | 1.0  nc  2.09  3.48  5.47  7.80 | 0.50-8.77  0.78-15.57  0.91-32.78  1.86-32.67 | 0.312  0.103  0.063  0.005 |
| **ypN Stage^d^** | **ypN0**  **ypN1**  **ypN2 ypN3** | 92 | 1.0  3.44  1.99  17.27 | 1.05-11.21  0.54-7.37  5.48-54.46 | 0.041  0.304  <0.001 | 92 | 1.0  3.85  1.49  18.75 | 1.16-12.86  0.32-7.05  5.82-60.43 | 0.028  0.614  <0.001 |
| **pCR Breast^d,e^**  **pCR Breast/Axilla^d,e^** |  | 93  93 | 5.34  6.66 | 1.57-18.15  1.54-28.58 | 0.007  0.011 | 93  93 | 4.68  5.90 | 1.36-16.06  1.36-25.56 | 0.014  0.018 |
| **Platinum-Based NACT^d^**  **Platinum-Based NACT^d,f^** |  | 91  91 | 0.49  0.34 | 0.14-1.74  0.08-1.5 | 0.273  0.153 | 91  NA | 0.54  NA | 0.15-1.93  NA | 0.347  NA |

n= number of patients; HR Hazard Ratio; CI, Confidence Interval; nc, not calculable; NACT, neoadjuvant chemotherapy; pCR, pathological complete response; NA, Not applicable; a, HR by Cox regression survival analysis; b, Basal status: any positivity for either cytokeratin 5/6 or EGFR by immunohistochemistry; c, Data for patients who did not receive NACT; d, Data for patients who received NACT; e, HR given for a non-pCR using pCR as the baseline value; f, Analysis limited to 24 months follow-up. Note: There was no association between the six tumour types and DFS or MFS (Data not shown)
