# Supplementary material for: Outcome for triple negative breast cancer in a retrospective cohort with an emphasis on response to platinum-based neoadjuvant therapy
Source: Breast Cancer Res Treat. 2018 Nov 28;174(1):1–13. doi: 10.1007/s10549-018-5066-6 (PMC6418073; doi:10.1007/s10549-018-5066-6)
Supplement: Supplementary file 2 — Supplementary material 2 (DOCX 16 KB) [file 10549_2018_5066_MOESM2_ESM.docx]

**Supplementary Table 2 Univariate Analysis of BCSS for all patients**

| **Parameter** |  | **n** | **HR^a^** | **95% CI** | **p-value** |
| --- | --- | --- | --- | --- | --- |
| **Age at Diagnosis** |  | 320 | 1.02 | 1.00-1.04 | 0.034 |
| **Menopausal Status** |  | 306 | 1.24 | 0.73-2.10 | 0.426 |
| **Tumour Grade (2 vs. 3)** |  | 315 | 0.85 | 0.47-1.54 | 0.595 |
| **Tumour Type** |  | 271 | 0.97 | 0.80-1.17 | 0.752 |
| **Basal Status^b^** |  | 300 | 1.06 | 0.57-1.99 | 0.852 |
| **pT Stage^c^** | **pT1**  **pT2**  **pT3 pT4** | 213 | 1.0  1.31  3.04  13.68 | 0.61-2.81  0.95-9.69  5.62-33.3 | 0.482  0.060  <0.001 |
| **pN Stage^c^** | **pN0**  **pN1**  **pN2**  **pN3** | 204 | 1.0  1.16  4.80  5.91 | 0.48-2.77  2.21-10.41  2.33-14.96 | 0.745  <0.001  <0.001 |
| **M1 at Diagnosis** |  | 338 | 9.66 | 5.29-17.65 | < 0.001 |
| **pCR Breast^d, e^**  **pCR Breast/Axilla^d, e^** |  | 96  96 | 4.11  10.30 | 1.19-14.21  1.37-77.38 | 0.025  0.023 |
| **ypT Stage^d^** | **pN0**  **ypTis**  **ypT1**  **ypT2**  **ypT3 ypT4** | 96 | 1.0  nc  2.13  1.61  5.20  6.89 | 0.51-8.91  0.27-9.63  0.87-31.18  1.72-27.60 | 0.302  0.603  0.071  0.006 |
| **ypN Stage^d^** | **yN0**  **ypN1**  **ypN2 ypN3** | 95 | 1.0  6.31  3.58  41.99 | 1.57-25.31  0.80-16.06  10.99-160.30 | 0.009  0.095  <0.001 |
| **Platinum-based therapy^d^** |  | 94 | 0.20 | 0.03-1.57 | 0.126 |

n= number of patients; HR Hazard Ratio; CI, Confidence Interval; nc, not calculable; M1, metastatic disease.

a, Cox regression survival analysis; b, Basal status: any positivity for either cytokeratin 5/6 or EGFR by immunohistochemistry; c, Data for patients who did not receive NACT; d, Data for patients who received NACT; e, HR given for a non-pCR using pCR as the baseline.
