# Supplementary material for: Outcome for triple negative breast cancer in a retrospective cohort with an emphasis on response to platinum-based neoadjuvant therapy
Source: Breast Cancer Res Treat. 2018 Nov 28;174(1):1–13. doi: 10.1007/s10549-018-5066-6 (PMC6418073; doi:10.1007/s10549-018-5066-6)
Supplement: Supplementary file 3 — Supplementary material 3 (DOCX 17 KB) [file 10549_2018_5066_MOESM3_ESM.docx]

**Supplementary Table 3 Multivariable analysis of DFS, MFS and BCSS adjusted for pCR Breast**

|  | **n** | **HR** ^a^ | **95% CI** | **p-value** |
| --- | --- | --- | --- | --- |
| **Disease Free Survival** | 85 |  |  |  |
| Age at Diagnosis  Tumour Grade  Tumour Type  Basal Status^b^  Platinum-based therapy  pCR Breast^c^ |  | 0.99  0.97  0.69  1.68  0.66  7.14 | 0.95-1.03  0.36-2.63  0.31-1.51  0.38-7.48  0.17-2.51  1.59-32.07 | 0.686  0.958  0.351  0.497  0.543  0.010 |
| **Metastasis Free Survival** | 85 |  |  |  |
| Age at Diagnosis  Tumour Grade  Tumour Type  Basal Status^b^  Platinum-based therapy  pCR Breast^c^ |  | 0.99  0.80  0.71  1.53  0.67  5.90 | 0.95-1.04  0.26-2.25  0.33-1.53  0.34-6.90  0.18-2.59  1.30-26.84 | 0.750  0.673  0.384  0.582  0.565  0.022 |
| **Breast Cancer Specific Survival** | 88 |  |  |  |
| Age at Diagnosis  Tumour Grade  Tumour Type  Basal Status^b^  Platinum-based therapy  pCR Breast^c^ |  | 1.01  0.94  0.80  1.45  0.27  4.97 | 0.96-1.06  0.32-2.71  0.44-1.48  0.31-6.73  0.03-2.18  1.11-22.24 | 0.738  0.906  0.476  0.638  0.219  0.036 |

n= number of patients; HR Hazard Ratio, Cox regression survival analysis; CI, Confidence Interval; pCR, pathological complete response.

a, Cox regression survival analysis; b, Basal status: any positivity for either cytokeratin 5/6 or EGFR by immunohistochemistry; c, HR given for a non-pCR using pCR as the baseline value.
