# Supplementary material for: Outcome for triple negative breast cancer in a retrospective cohort with an emphasis on response to platinum-based neoadjuvant therapy
Source: Breast Cancer Res Treat. 2018 Nov 28;174(1):1–13. doi: 10.1007/s10549-018-5066-6 (PMC6418073; doi:10.1007/s10549-018-5066-6)
Supplement: Supplementary file 4 — Supplementary material 4 (DOCX 15 KB) [file 10549_2018_5066_MOESM4_ESM.docx]

**Supplementary Table 4 Multivariable analysis of DFS at 24 months in patients who received NACT**

|  | **n** | **HR^a^** | **p-value** | **95% CI** |
| --- | --- | --- | --- | --- |
| **Disease Free Survival** | 90 |  |  |  |
| Age at Diagnosis  Tumour Grade  Platinum-based therapy  ypT  ypN |  | 0.91  1.57  0.60  1.10  3.69 | 0.015  0.468  0.521  0.681  0.000 | 0.84-0.98  0.47-5.30  0.12-2.88  0.71-1.71  1.88-7.25 |

n= number of patients; HR Hazard Ratio given for non-pCR with pCR as a baseline value; CI, Confidence Interval.

a, Cox regression survival analysis
